# *InVivo* Molecular Ultrasound Assessment of Glioblastoma Neovasculature with Endoglin-Targeted Microbubbles

**DOI:** 10.1155/2018/8425495

**Published:** 2018-10-18

**Authors:** Cheng Liu, Fei Yan, Yajie Xu, Hairong Zheng, Lei Sun

**Affiliations:** ^1^Department of Biomedical Engineering, The Hong Kong Polytechnic University, Hong Kong SAR, China; ^2^Paul C. Lauterbur Research Center for Biomedical Imaging, Shenzhen Institutes of Advanced Technology, Chinese Academy of Sciences, Shenzhen, China

## Abstract

**Objectives:**

Glioblastoma, as one of the most malignant cancer in the world, usually shows substantially increased angiogenesis. Endoglin (CD105), which is an alternative proangiogenic growth factor, has been remarkably upregulated on the proliferating glioblastoma neovasculature. However, little is known on the noninvasive assessment of the expression levels of CD105 during glioblastoma progression. Herein, we investigated the potential of the molecular ultrasound imaging for the noninvasive assessment of the expression levels of the biomarker CD105 during the glioblastoma progression.

**Materials and Methods:**

The CD105-targeted perfluorocarbon-containing lipid-shelled microbubbles (MBs) were prepared. A parallel flow chamber was employed, in which the CD105-targeted and non-targeted MBs were tested across the CD105 ± expression cell lines. *In vivo* molecular US imaging was conducted based on a subcutaneous xenograft tumor model (*n*=9). Finally, the statistical analysis was conducted to quantitatively correlate the attachment numbers of MBs in the parallel flow chamber test with the CD105 expression levels of the cells in the flow cytometry test and the *in vivo* molecular ultrasound signals with the *ex vivo* expression levels of CD105 in the immunohistochemical test.

**Results and Discussion:**

The attachment numbers of the CD105-targeted MBs significantly correlated with the CD105 expression levels of the cells in the parallel flow chamber test. There was a good correlation between the *in vivo* molecular ultrasound signals with the CD105-targeted MBs and the *ex vivo* expression levels of CD105 in the immunohistochemical test. The results indicate that the molecular US imaging is much potential to assess the progression of the glioblastoma neovasculature noninvasively.

## 1. Introduction

Glioblastoma, which is one of the most malignant cancer types of the central nervous system, continues to cause high morbidity and mortality rates in the world [1]. Although significant development has been made in the glioblastoma management, various challenges still remain, such as diagnosis at the early stage [2]. At the early progression stage of glioblastoma, the production of neovasculature (blood vessels) from the preexisting vessels (mature) or microvessels is essential to the pathological processes, which provides the oxygen and nutrients to the malignant cells for rapid growth. Typically, after reaching a certain size, the malignant cells enter the exponential growth phase, during which the realignment and distributions of the endothelial cells lead to the neovasculatures around and within the malignant cells [3].

The formation of neovasculature is a complicated process with multiple steps, which is promoted by a series of proangiogenic growth factors (e.g., VEGF) [4]. These proangiogenic growth factors can work as the biomarkers of molecular imaging for the noninvasive assessment of the tumor progression. Among those proangiogenic biomarkers, endoglin (CD105) has been shown to be remarkably upregulated on highly proliferating endothelial cells (neovasculature wall), instead of the normal endothelial cells or mature vessels [5, 6]. In clinic, it is reported that the outcome of the anticancer therapies targeting VEGF have not met the high expectation, which could be due to the over expression of the alternative proangiogenic growth factor (e.g., endoglin (CD105)). Besides, pathologists have been using endoglin as an independent prognostic target for the assessment of the aggressiveness of most solid tumor types [7, 8]. Thus, the endoglin (CD105) has drawn a lot of attention as a novel alternative biomarker for the tumor diagnosis, prognosis, and therapy. Several preliminary studies have indicated endoglin (CD105) as a potential biomarker for different molecular imaging strategies, such as single-photon emission computed tomography (SPECT) [9], magnetic resonance imaging (MRI) [10], near-infrared fluorescence imaging [11], and ultrasound imaging [12]. However, little is known on the assessment of the expression levels of endoglin (CD105) during the glioblastoma progression *in vivo*. Understanding of the temporal and spatial expression levels of endoglin (CD105) *in vivo* could potentially contribute greatly to both the early diagnosis and anticancer therapy of glioblastoma.

Ultrasound imaging (US) is a popular imaging tool that utilizes unique acoustic-tissue interface behavior of the sound waves as it passes through a biological organ or tissue of interest. Ultrasound imaging is widely used due to its uniqueness that can be used for applications both in the diagnosis and therapy [13, 14]. Conventional ultrasound imaging has been well accepted as an imaging modality specialized for the morphological and functional imaging. While molecular ultrasound imaging, which employs functionalized contrast agents, is potentially capable to assess the tumor angiogenesis noninvasively and quantitatively *in vivo* [15]. Recently, microbubbles (MBs), which are liquid shell emulsions filled with gas (e.g., perfluorocarbon, nitrogen, sulfur hexafluoride, or air), have been used as contrast agents for molecular ultrasound imaging [16]. The shells of the microbubbles are usually composed of materials with good biocompatibility (e.g., lipid, protein, and polymers). The structure of MBs makes it unique in resonating and sending back high nonlinear harmonic and subharmonic ultrasound signals when exposed in the ultrasound mechanical waves, which would bring about high contrast-to-background ratio [17]. Importantly, the size of microbubbles usually in 1∼4 *µ*m in diameter would limit them from going extravascularly from the blood vessels, that makes them quite suitable for the vasculature-related applications *in vivo* [18].

**Figure 1:**
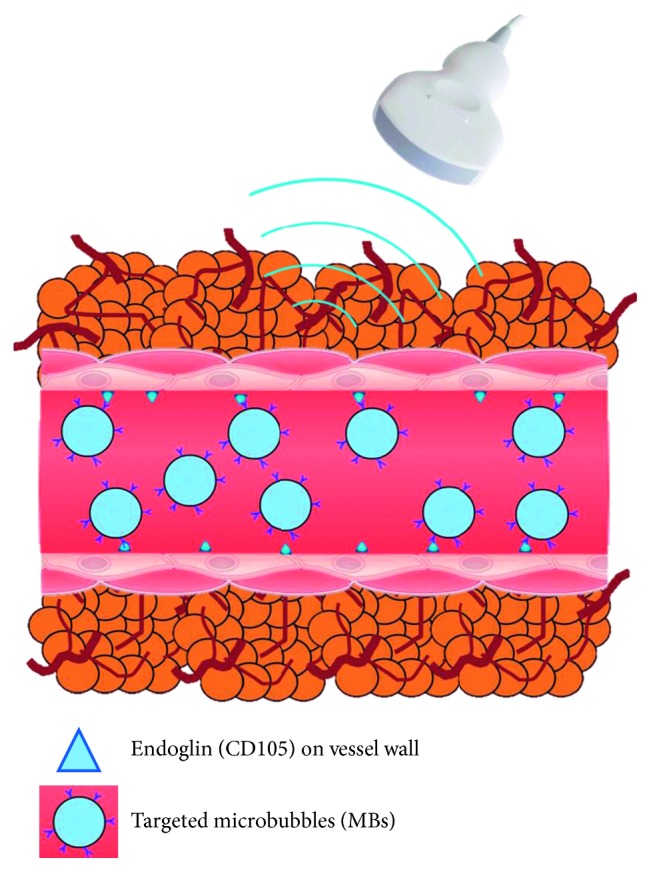
Illustration of the molecular ultrasound imaging strategy. Noninvasive assessment of the expression levels of the alternative proangiogenic growth factor endoglin (CD105) on the vessel wall by using the CD105-targeted microbubbles (MBs) with 1∼4 *µ*m in diameter.

Microbubbles (MBs) are usually functionalized with ligands such as antibodies or peptides that bind the biomarkers of interest with high affinity [15]. Several studies have validated the use of MBs to detect the tumor angiogenesis in animal models by targeting to the proangiogenic growth biomarkers [19–21]. It is shown that targeted MBs could accumulate more in the tumor regions than the nontargeted MBs, which would significantly increase the ultrasound response strength. The aim of this study is to develop the CD105-targeted MBs and investigate the potential of molecular US for the noninvasive assessment of the expression levels of endoglin (CD105) during glioblastoma progression from small to large sizes *in vivo*, as illustrated in Figure 1.

## 2. Materials and Methods

### 2.1. Synthesis and Characterization of CD105-Targeted MBs

**Figure 2:**
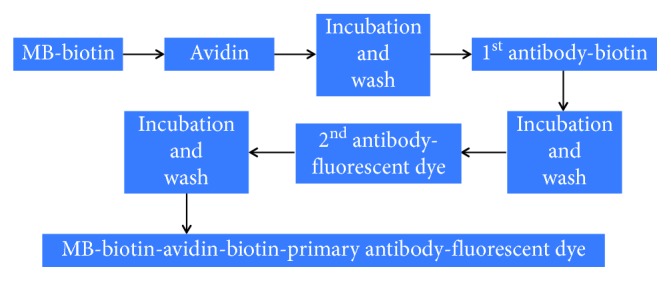
Preparation procedures of the microbubbles (MBs). Two types of MBs were prepared: (1) CD105-targeted MBs as MBs-biotin-avidin-biotin-CD105-biotin and (2) control nontargeted MBs as MBs-biotin-avidin-biotin-IgG-biotin. Biotinylated rat antimouse CD105 monoclonal antibodies (eBioscience) and biotinylated rat immunoglobulin G antibodies (IgG) were used as 1^st^ antibodies for the CD105-targeted MBs and the control nontargeted MBs, respectively. The 2^nd^ antibodies, which were fluorescein-conjugated antibiotin antibodies (Jackson ImmunoResearch), were used to confirm the affinity of the 1^st^ antibodies on the shell of MBs through the specific biotin-avidin conjugation.

Two types of microbubbles (MBs), including (1) CD105-targeted MBs and (2) nontargeted MBs with an isotype-matched control immunoglobulin G antibody, were prepared by using streptavidin-biotin binding chemistry according to a reported protocol [22, 23]. The perfluorocarbon-containing lipid-shelled MBs-containing streptavidin moieties in the lipid shell were reconstituted in 1 mL sterile saline (0.9% sodium chloride), which are abbreviated as MB-biotin in Figure 2. For targeting the MBs-biotin, 5 *μ*g of the following two types of 1^st^ antibodies were incubated with 5 × 10^7^ avidin-conjugated MBs-biotin for 10 mins at room temperature, respectively: (1) biotinylated rat antimouse CD105 monoclonal antibodies (eBioscience), which has been reported for use in flow cytometry analysis/cell sorting for specific targeting with the mouse endoglin (CD105) molecule [24, 25] and (2) biotinylated rat control immunoglobulin G antibodies (IgG) (eBioscience). The 2^nd^ antibodies, which were fluorescein-conjugated antibiotin antibodies (Jackson ImmunoResearch), were used to confirm the affinity of the 1^st^ antibodies on the shell of MBs through the specific biotin-avidin binding chemistry, as shown in Figure 2. The free avidin and antibodies were removed by washing with PBS. The mean and standard deviation of the MBs' diameter were assessed by an optical particle counter with a 0.5 mm diameter detection limit (AccuSizer 780; Particle Sizing Systems, Santa Barbara, CA, USA).

### 2.2. Parallel Flow Chamber Test

To assess the binding specificity of the CD105-targeted MBs to the biomarker CD105, the parallel flow chamber test was performed, according to a reported protocol [26].

Two types of cell lines, mouse endothelial cell line MS1 and mouse breast cancer cell line 4T1, were selected as the cell lines with high and low CD105 expression levels, respectively. Both the MS1 and 4T1 cell lines were purchased from the National Infrastructure of Cell Line Resource (Chinese Academy of Science, Shanghai, China). For the MS1 cells, the culture medium was ATCC-formulated Dulbecco's modified Eagle medium (ATCC) with 5% fetal bovine serum and 1% penicillin-streptomycin. For the 4T1 cells, they were cultured in ATCC-formulated RPMI-1640 medium with 10% fetal bovine serum and 1% penicillin-streptomycin. Both cell lines were cultured in sterilized environment with 5% CO_2_ humidified condition and 37°C air atmosphere.

**Figure 3:**
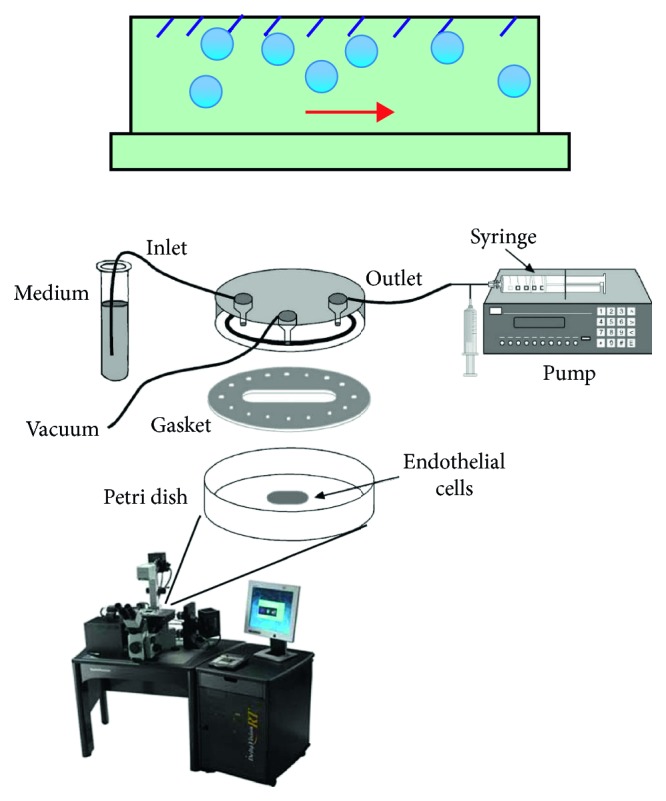
Illustration of the parallel flow chamber test. The cells were coated on the bottom of Petri dish, while the MBs would flow through the chamber as the red arrow shows. Two types of cell lines, including MS1 and 4T1, were selected as cell lines with high and low CD105 expressing levels, respectively. The CD105-targeted MBs and nontargeted MBs would be used to test the attachment specificity in the absence and presence of blocking antibodies.

The cell culture dishes were pretreated with 50 *µ*g/ml collagen (type 1, rat tail, BD Biosciences, Bedford, MA) in 0.02 M acetic acid for 1 hr, aspirated and rinsed with sterile DPBS prior to the cell coating. Two million MS1 cells and two million 4T1 cells were coated on different cell culture dishes, respectively. The dishes would then be tested on the parallel flow chamber, as shown in Figure 3. The solutions would be passed over the two types of cells in the parallel flow chamber in the following order: (a) PBS, (b) 5 × 10^7^ of CD105-targeted MBs and control MBs in PBS, and (c) PBS. Afterwards, the dishes would then be imaged immediately with dark-field microscopy. Six random optical fields of view per dish would be selected for the quantification of the number of attached MBs per cell. In order to further confirm the binding specificity of the CD105-targeted MBs, another two groups of cells, including MS1 and 4T1, were incubated with antimouse CD105 monoclonal antibodies to block the CD105 receptor in prior, followed by the parallel flow chamber test. Triplicate repetitions were performed.

### 2.3. Subcutaneous Tumor Model

All procedures using laboratory animals were approved by the Department of Health, the Government of the Hong Kong Special Administrative Region, and the Hong Kong Polytechnic University Animal Subjects Ethics Sub-Committee. Tumors were established by subcutaneous injection of 5 × 10^6^ U-87 MG glioblastoma cells dissolved in 50 *µ*L suspension (National Infrastructure of Cell Line Resource, Chinese Academy of Science, Shanghai, China) into the right hind limb of 6–8-week old female nude mice [27]. A total of 9 pieces of tumors were produced and used in this study. Tumor volumes of each mouse were measured and recorded daily with B-mode ultrasound imaging. According to the tumor volumes, the mice were divided into 3 groups: 50–150 mm^3^ as small group, 151–250 mm^3^ as medium group, and larger than 250 mm^3^ as large group. All tumors were scanned using Vevo2100 high-frequency ultrasound system (FUJIFILM VisualSonics, Toronto, Canada) with an LZ-250 linear array transducer (center frequency at 21 MHz, 256 elements, lateral and axial resolution of 165 and ∼75 *µ*m, respectively, maximum imaging depth of 20 mm).

### 2.4. *In Vivo* Molecular Ultrasound Imaging Experiment

All the mice would be kept under anesthesia with 2% isoflurane in room air during the experiment, and all the imaging experiment settings would be kept constant throughout the imaging experiment. Molecular ultrasound imaging was performed using Vevo2100 high-frequency ultrasound system (FUJIFILM VisualSonics, Toronto, Canada) with an LZ-250 linear array transducer. The central planes of those tumors would be aligned by the guidance of the B-mode ultrasound imaging.

**Figure 4:**
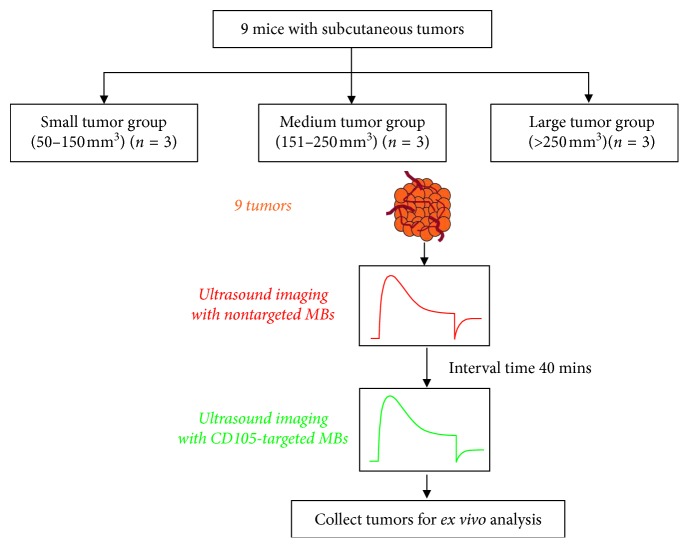
The experiment design of *in vivo* molecular US imaging experiment. Subcutaneous tumor models were established for the assessment of CD105 expression levels during tumor progression from small to large size. The nontargeted MBs were injected and measured as the control, and the CD105-targeted MBs were injected with a 40 min interval after the injection of nontargeted MBs. The US signals from the CD105-targeted MBs would be calculated by averaging predestruction and postdestruction imaging signals and subtracting the postdestruction signal average from the predestruction signal average.

Since the ultrasound signal contribution from the attached CD105-targeted MBs also would depend on the regional MBs perfusion, the results representing the attached CD105-targeted MBs would need to be normalized to the blood flow perfusion reference condition measured by using the nontargeted MBs in the same animal before the CD105-targeted MBs imaging session, with the same imaging conditions, for all mice [28]. To allow full clearance of the nontargeted MBs from the previous imaging session, a 40  min interval was applied before the CD105-targeted MBs-imaging session to avoid any interference between the two sessions. This interval period of time between the two MBs injection was chosen according to a previously reported protocol [29]. It was demonstrated that most of the MBs would be cleared from the vasculature in 40 mins after I.V. injection. In all mice, data acquisitions were performed by injecting the two types of MBs in the following order: (1) control nontargeted MBs and (2) CD105-targeted MBs, into the same animal with an interval time of 40 mins, as shown in Figure 4.

**Figure 5:**
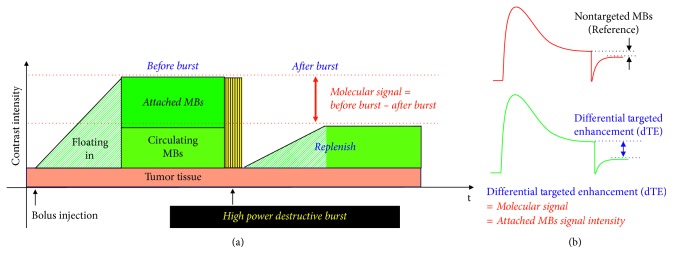
Principal of the quantitative assessment of molecular US signals. (a) Burst-and-replenishment technique for the quantitative assessment of the attached MBs; (b) the differential targeted enhancement (dTE) would be used to indicate the molecular US signal which is contributed by attached MBs only. The nontargeted MBs were used as a reference for the CD105-targeted MBs.

After injection of the microbubbles (MBs), molecular ultrasound imaging based on the ultrasound burst-and-replenish technique would be performed according to a reported protocol [30, 31]: 3 mins after the MBs injection, both B-mode ultrasound imaging and nonlinear contrast ultrasound imaging frames would be acquired and overlaid over a 10-second period for 250 frames. After that, a destruction burst (10 MHz; mechanical index, approximately 0.235) would be applied for 5 seconds to destroy all the MBs in the tumor region. After the destruction burst, another 250 frames would be acquired to record the replenishment procedure of the floating-in MBs, as shown in Figure 5(a). The regions of interest (ROI) within the tumors would be selected by an experienced reader. The molecular ultrasound signals from the CD105-targeted MBs would be calculated by averaging the predestruction and postdestruction imaging signals and subtracting the postdestruction signal average from the predestruction signal average, which was defined as differential targeted enhancement (dTE), as shown in Figure 5(b). This factor would be used to represent the molecular signal which is contributed by attached MBs. The molecular US images representing the attached MBs would be combined with the B-mode ultrasound anatomic images. Finally, the quantitative molecular US signals would be correlated with the result of *ex vivo* immunohistochemistry analysis (e.g., CD31 and CD105).

### 2.5. Statistical Analysis

The date would be analyzed and output as means ± standard deviations. For the parallel flow chamber test, a paired Wilcoxon test would be applied to compare the attachment number of CD105-targeted MBs with control non-targeted MBs, which passed over the two cell lines, including MS1 and 4T1. The different attachment numbers before and after the blocking with antibody would be tested with a paired Wilcoxon test. Also, a Spearman rank correlation (*ρ* values) would be used to test the correlation between the CD105 expression levels of the two types of cell lines assessed by the flow cytometry test and the attachment numbers of MBs in the parallel flow chamber test. Spearman rank correlation was applied to test the *in vivo* CD105-targeted US imaging results with the *ex vivo* immunofluorescence results in the three tumor groups. *P* < 0.05 would be considered to be a statistically significantly difference.

## 3. Results and Discussion

### 3.1. Parallel Flow Chamber Test of CD105-Targeted MBs *In Vitro*

**Figure 6:**
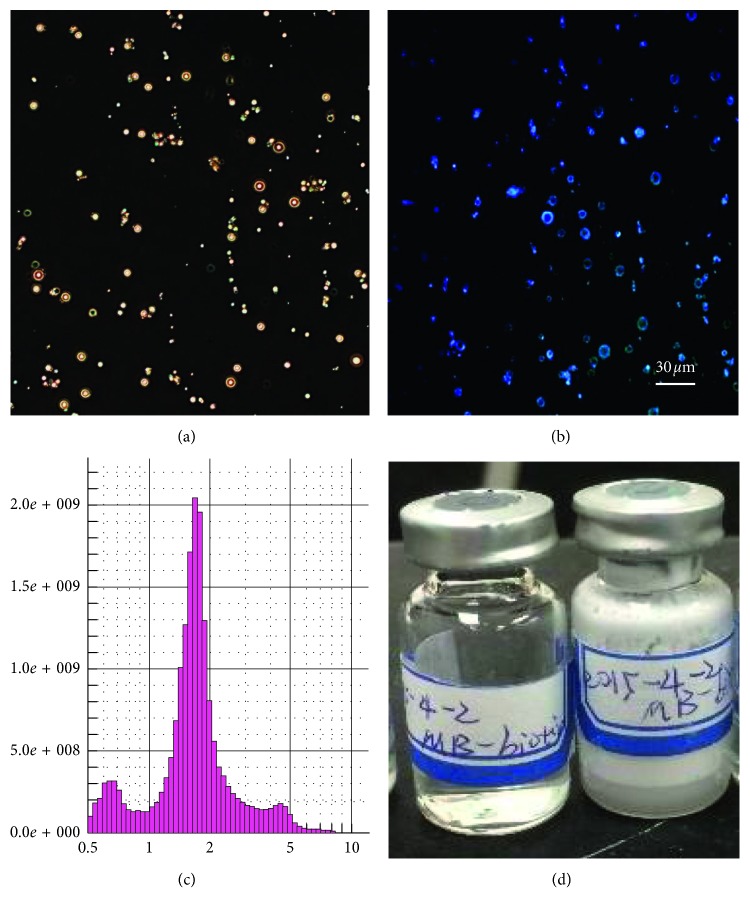
*In vitro* characterization of CD105-targeted MBs (MBs-biotin-avidin-biotin-anti-CD105-FITC). (a) The dark-field microscope image; (b) the fluorescence microscope image; (c) the size distribution of MBs, mean diameter in 2–3 *µ*m; (d) the photograph of prepared MBs kept in vials.

The morphology and size distribution of CD105-targeted MBs are shown in Figure 6. The fluorescence microscope imaging confirmed that the antimouse CD105 monoclonal antibodies were successfully bond to the shell of the MBs.

**Figure 7:**
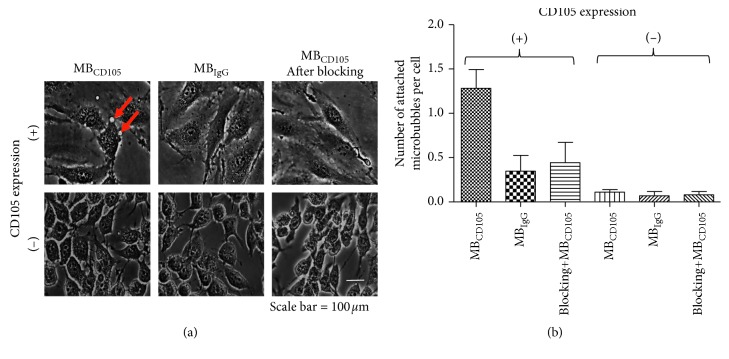
The parallel flow chamber test results. (a) Bright-field microscope images of the parallel flow chamber test. Two types of cell lines were used: endothelial cell MS1 with CD105 high expression level and 4T1 cells with CD105 low expression level. The CD105-targeted MBs and nontargeted MBs were used as control, while the anti-CD105 monoclonal antibody was used as the blocking control for CD105 high expressing cells. The red arrow indicates the location of the attached CD105-targeted MBs. The round small spots under the bright-field microscopy were MBs that were in contact with the membrane of cells without free floating movement. (b) The quantitative attachment results of the parallel flow chamber test. The attachment of the control nontargeted MBs to the MS1 cells was significantly (*P*=0.025) lower in comparison with the CD105-targeted MBs; The MS1 cells (CD105 positive) with blocking treatment would result in a significant (*P*=0.015) reduction in the attachment number of the CD105-targeted MBs, which could confirm the attachment specificity of the CD105-targeted MBs to the specific biomarker in the parallel flow chamber test.

The attachment number of the CD105-targeted MBs to MS1 cells (CD105 positive) was significantly (*P*=0.005) higher than that to 4T1 cells (CD105 negative), as shown in Figure 7. And, the attachment number of nontargeted MBs to MS1 cells was significantly (*P*=0.025) lower in comparison with the CD105-targeted MBs, as shown in Figure 7. Furthermore, the MS1 cells with the blocking treatment would result in a significant (*P*=0.015) reduction in the attachment number of the CD105-targeted MBs, as shown in Figure 7, which could confirm the attachment specificity of the CD105-targeted MBs to the specific biomarker in the parallel flow chamber test. Furthermore, the analysis between the attachment numbers of CD105-targeted MBs and the expression levels of CD105 in two types of cells as assessed by the flow cytometry test showed a significant positive correlation (*ρ*=0.76, *P* < 0.032).

### 3.2. *In Vivo* Assessment of Endoglin Expression Levels

**Figure 8:**
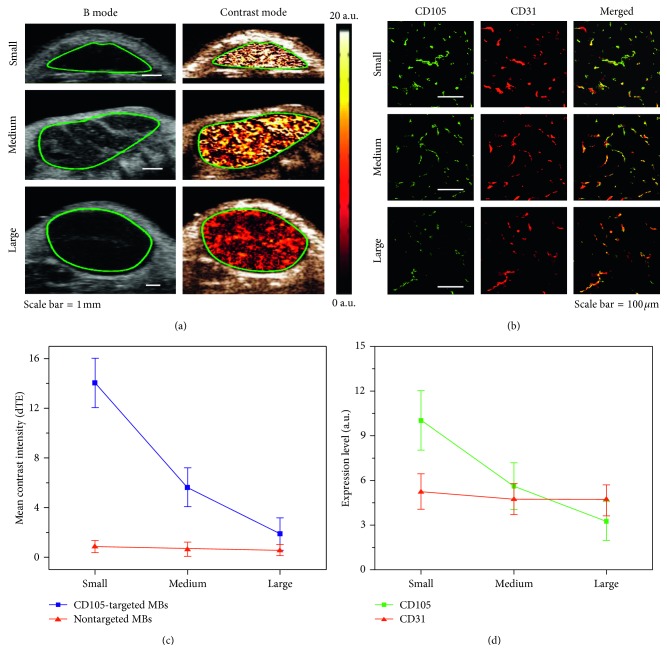
The *in vivo* assessment of the CD105 expression levels in the subcutaneous glioblastoma xenograft tumors (small, medium, and large sizes). (a) The dual-mode US imaging (B-mode and contrast mode) of tumors by using the CD105-targeted MBs, scale bar = 1 mm; (b) the *ex vivo* immunohistochemistry (IHC) test (e.g., CD105 and CD31) of the tumor tissues. CD105 was confirmed to be expressed on the endothelial cells; (c) the differential targeted enhancement (dTE) measured in the small, medium, and large glioblastoma tumor groups by using the CD105-targeted MBs and nontargeted MBs; (d) the qualification of the expression levels of CD105 and CD31 in tumor tissues assessed by *ex vivo* immunohistochemistry (IHC). The statistical results indicated that the expression levels of endoglin (CD105) in the small and medium size tumors were significantly higher (*P* < 0.032) in comparison with CD31. And in large size tumors, the expression levels of endoglin (CD105) were significantly lower (*P* < 0.023) than those of CD31.

There was a good positive correlation between the *in vivo* molecular US signal and *ex vivo* expression levels of CD105 as assessed with the immunofluorescence test (*ρ*=0.86, *P* < 0.001), as shown in Figure 8. Additionally, the immunofluorescence test confirmed that the expressions of both CD105 and CD31 were colocalized on the endothelial cells, as shown in Figure 8, which demonstrated that the *in vivo* molecular US signals specifically came from those attached CD105-targeted MBs on the endothelial cells. In the subcutaneous tumor model, the expression levels of endoglin (CD105) in small and medium size tumors were significantly higher (*P* < 0.032) in comparison with CD31. In large size tumors, the expression levels of endoglin (CD105) was significantly lower (*P* < 0.023) than those of CD31, as shown in Figure 8.

### 3.3. Discussion

Ultrasound imaging is a popular imaging tool that utilizes unique acoustic-tissue interface behavior of sound waves at high frequency as it passes through a biological organ or tissue of interest. Ultrasound is widely used due to its uniqueness that can be used for applications in both diagnosis and therapy. Conventional ultrasound has been well accepted as an imaging modality specialized for morphological imaging. The advantages of ultrasound include, but not limited to, economic cost, availability, portability, high temporal resolution, ionizing radiation free, and high sensitivity. However, ultrasound imaging is not competent in imaging structures containing bone or air, because ultrasound waves could not transmit bone or air.

Molecular ultrasound imaging, which employs functionalized ultrasound contrast agent, is potentially able to assess tumor angiogenesis noninvasively and quantitatively. Recently, ultrasound contrast agents are based on microbubbles (MBs), which are liquid shell emulsions filled with gas, such as perfluorocarbon, sulfur hexafluoride, and nitrogen. The structure of microbubbles (MBs) makes it unique in very high echogenic response when exposed in ultrasound mechanical waves. On the one hand, this mechanical echogenic response would bring about high contrast-to-background ratio. On the other hand, the size of microbubbles usually 1∼4 *µ*m in diameter could limit them from going to extravascular regions. Overall, molecular ultrasound imaging with the aid of MBs is quite potential for detecting biomarkers that are overexpressed on the vessel wall. Among all the contrast agent-centered imaging modalities, US molecular imaging is a recently emerging one in the preclinical translation phase, whose clinical potential might be fully exploited in the next decade.

In this study, the expression levels of CD105, which are highly relevant to the growth of glioblastoma neovasculature, have been assessed by the molecular US imaging *in vivo* based on a subcutaneous tumor model. The *in vivo* US signals from the CD105-targeted MBs have shown that the expression levels of CD105 decreased when the tumor progressed to a larger size which was correlated with the histology result (costaining CD105/CD31) *ex vivo.* The *in vitro* binding test showed that the CD105-targeted MBs could target to a specific biomarker (CD105) on the positive CD105 expression cells. In this study, two types of cell lines (MS1 and 4T1) were selected as the CD105 ± expression cells for the assessment of the expression levels of CD105 in the parallel flow chamber test. The *in vitro* parallel flow chamber test was used as a mimic situation for the *in vivo* blood flow environment, which could be used to test the binding affinity of the CD105-targeted MBs *in vitro.* The parallel flow chamber test showed that the binding affinity and specificity were adequate for *in vivo* applications. And, the successful binding of avidin-biotin conjugate was tested using the fluorescein-conjugated antibiotin antibodies under the visualization of fluorescence microscope. Overall, the molecular US imaging as a preclinical research tool was used to evaluate the expression levels of neovasculature-related endoglin (CD105) *in vivo* on the glioblastoma subcutaneous xenograft model. The statistical results *in vivo* and the *in vitro* parallel flow chamber test together have validated this strategy as a noninvasive method to assess the progression of neovasculature for glioblastoma *in vivo.*

The assessment of tumor angiogenesis is one of the most popular applications of molecular US imaging. Among all the proangiogenic growth factors, vascular endothelial growth factor (VEGF) is the best-studied one and has gained much expectation for clinical translation. Besides VEGF, endoglin (CD105) acts as an alternative proangiogenic growth factor. In clinic, the endoglin (CD105)-based immunohistochemistry test is accepted as a standard test to assess the tumor angiogenesis by quantifying the microvessel density (MVD) for many types of solid tumors [32, 33]. It has been approved that endoglin (CD105) could be selectively expressed on the highly proliferating endothelial cells rather than the normal and mature endothelial cells. However, the studies of molecular US imaging that are relevant to the alternative proangiogenic growth factor endoglin (CD105) are still in the developing phase according to the literature [34].

The noninvasive molecuar US imaging and assessment of endoglin (CD105) are potential to act as an alternative strategy for monitoring tumor angiogenesis. Therefore, we are motivated to develop the CD105-targeted molecular US imaging strategy for the assessment of glioblastoma neovasculature. The CD105-targeted US contrast agent (microbubbles) has been investigated in solution, *in vitro* parallel flow chamber test, and *in vivo* subcutaneous tumor model. Noteworthily, the micrometer size microbubbles would be limited within the blood lumen, which are quite suitable for applications to the intravasculature biomarkers. The targeted microbubbles, when decorated with the functional ligands, could actively bind to the highly expressed endoglin (CD105) on the tumor neovasculature. Because the US signal intensity and harmonic components from the MBs are substantially higher and richer than the signals from the surrounding tissues, the targeted MBs that are accumulated in the neovasculature can be identified and visualized with the molecular US imaging with high sensitivity. Although the image resolution of molecular US image was limited to delineate the morphology of the biomarker CD105 expression *in vivo*, the quantitative molecular US signals were demonstrated to be correlated with the IHC analysis *ex vivo.* In addition, the molecular US images representing the attached MBs to biomarker CD105 in the molecular level could be combined with the complementary B-mode ultrasound anatomic images *in vivo*.

## 4. Conclusions

Among all the contrast agent-centered imaging modalities, molecular ultrasound imaging is a recently emerging one in the preclinical translation phase, whose clinical potential could be fully exploited in the next decade. Endoglin (CD105), as an alternative angiogenic factor on the luminal surface of glioblastoma neovasculature, is suitable to work as the binding target of ultrasound contrast agent. Molecular US imaging with the aid of targeted MBs is suitable for assessing the neovasculature progression of glioblastoma at the early stage by visualization of the proangiogenic biomarker endoglin (CD105), which is highly expressed on the neovasculature wall. This study is a proof of concept work which may develop towards preclinical translation in the future.

## Data Availability

The data used to support the findings of this study are available from the corresponding author upon request.

## Supplementary Materials

Supplementary Materials Video of in vivo “burst-and-replenishment” experiment for quantitative assessment of the CD105 expression level with CD105-targeted MBs.
